# Tele-support in breastfeeding: position statement of the Italian society of Neonatology

**DOI:** 10.1186/s13052-024-01816-5

**Published:** 2024-11-09

**Authors:** Riccardo Davanzo, Maria Enrica Bettinelli, Mariella Baldassarre, Isabella Mondello, Antonella Soldi, Silvia Perugi, Maria Lorella Giannì, Lorenzo Colombo, Guglielmo Salvatori, Laura Travan, Giuseppe Giordano, Breastfeeding Section of the Italian Society of Neonatology

**Affiliations:** 1 Nutrition Research Centre, University of Insubria, Varese, Italy; 2 ASST FBF Sacco, Attività Consultoriali, Milan, Italy; 3 Neonatologia e TIN Policlinico, Bari, Italy; 4 Neonatologia e TIN, Grande Ospedale Metropolitano, Reggio Calabria, Italy; 5 Neonatologia Universitaria Ospedale Sant’Anna, Turin, Italy; 6 Neonatologia AOU Careggi, Florence, Italy; 7 NICU, Fondazione IRCCS Cà Granda Ospedale Maggiore Policlinico di Milano, Milan, Italy; 8 Dipartimento Di Scienze Cliniche E Di Comunità, Università degli Studi di MIiano, Milan, Italy; 9 Neonatal Intensive Care Unit - “Bambino Gesù” Children’s Hospital IRCCS, Rome, Italy; 10 Division of Neonatology, Institute for Maternal and Child Health, IRCCS “Burlo Garofolo”, Trieste, Italy; 11 Neonatologia e TIN, Ospedali Riuniti Villa Sofia-Cervello, Palermo, Italy

**Keywords:** Tele-support, Breastfeeding, Position statement, Focus group, Survey, Maternity Hospitals

## Abstract

Tele-support in breastfeeding can be defined as any support provided by a service that connects health workers and/or lactation consultants with breastfeeding parents through video visits, although a telephone contact with the breastfeeding mother remains the first method of remote breastfeeding support. The tele-support in breastfeeding has increased significantly during the SARS-CoV2 pandemic worldwide and, given its effectiveness, may be maintained also after the pandemic. The Italian Society of Neonatology on the basis of: 1) two focus group studies on the tele-support in breastfeeding conducted in Italy with 11 Neonatal Intensive Care Unit nurses and 10 neonatologists, respectively, 2) a national survey on tele-support in breastfeeding addressing the Italian Neonatal Intensive Care Units, and 3) a review of the available experiences and literature, has provided a Position Statement, limitedly to the individual tele-support in breastfeeding. The Italian Society of Neonatology states that: 1) the tele-support in breastfeeding can be used when a consultation in person is not shortly available and may allow to select those situations that require an in-person visit; 2) the organization of a tele-support in breastfeeding session requires the use of a competent, dedicated healthcare staff (specifically trained and/or with adequate experience) and an appropriate methodology while preparing, running and concluding the support session. According to Italian Society of Neonatology the tele-support in breastfeeding may be an effective intervention to promote breastfeeding as a complementary method to the in-person assistance and should be possibly provided in an integrated manner by the Community Health Services and the Maternity Hospital.

## Introduction

The initial concept of telemedicine defined by Bird in 1975 [1] was expanded by the World Health Organization (WHO) to that of telehealth, that implies «the provision of health services, when distance is a critical factor, by any health worker, who uses information and communication technologies for the exchange of information useful for diagnosis, treatment, prevention, research and continuous training" [2]. The more recent term eHealth (“e” for electronic) underlines the complexity of the need for network resources and information technologies [3]. Finally, mHealth (“m” for mobile) focuses on healthcare practices supported by mobile communication tools (smartphones, tablets, smartwatches, implantable devices, wearable devices, iPads) that, according to WHO, are expected to increase universal healthcare coverage, with synchronous (video calls, video conferences, Facebook) or asynchronous (text messages, apps, online platforms) modes [4–6].

The use of telemedicine increased significantly during the SARS-CoV2 pandemic worldwide, making it possible to reduce transmission of COVID-19 while providing some form of continuity of care. The community of health professionals had to suddenly grapple with the use of available, in a way sometimes unfamiliar, technologies, understanding their potential benefits, applicability and even limitations [7]. Finally, telemedicine seemed to represent a useful aid in providing healthcare, possibly to be maintained and efficiently integrated into the healthcare system.

## eHealth in pediatric and neonatal care

In the face of little qualitative and quantitative research on telemedicine in the pediatric age [8], specific consensus documents have been drawn up in Italy [9–11], setting standards of care in particular for medically complex children with chronic diseases.

When addressing the perinatal and neonatal field, telemedicine can be applied to a wide range of situations, from fetal medicine to resuscitation in the delivery room, ventilated newborns, follow-up of high-risk neonates discharged from the Neonatal Intensive Care Unit (NICUs). Telemedicine less often concerns the tele-support on breastfeeding (TSB) [4, 9, 12], that can be defined as any support provided by a service that connects health workers (midwives, nurses and physicians) and/or lactation consultants with breastfeeding parents through video visits.

In the context of this relatively wide range of potential implementations in the perinatal and neonatal field, an evaluation system called Supporting Pediatric Research in Outcomes and Utilization of Telehealth (SPROUT) has been suggested, in order to assess the usefulness of telemedicine in providing sustainable and quality care. It is recognized that an effective and sustainable telemedicine service requires adequate planning, taking into account that the codified practices in use for in-person healthcare may need to be adapted for remote provision [11]. In fact, SPROUTS supports the preliminary identification of the outcome, the definition of the extent of its improvement and the related costs [4]. Although SPROUT refers in particular to the NICU context, it can also usefully guide in the context of Tele-support in Breastfeeding (TSB).

Despite the awareness that the terms Tele-medicine, Tele-health, eHealth and mHealth apply to different types of services and connectivity systems, in the context of TSB they may be used interchangeably.

In the present paper, we focus on the Italian experiences of TSB mainly in the neonatal period, and illustrate the ad hoc Position Statement of the Italian Society of Neonatology (SIN).

## Effectiveness of the TSB

A relevant experience on the TSB during COVID-19 pandemic has been done internationally, particularly in the United Kingdom, as mainly documented by two UNICEF-UK surveys conducted in 2020 [13, 14].

In April 2020, a first survey on 274 health workers, who deal with infant feeding and belong to the National Infant Feeding Network (NIFN)(https://www.unicef.org.uk/babyfriendly/wp- content/uploads/sites/2/2021/11/Summary-of-results-1-and-2-Infant-feeding-during-Covid-19.pdf), evaluated the impact of care during the COVID-19 period. The interviewees reported the redistribution of healthcare personnel and the reduction in staff and peer support staff in the perinatal care services. In this emergency situation, parents' access to perinatal care services was very limited or suspended in most cases and breastfeeding assistance was maintained in person only in approximately 10% of cases. The sudden decrease in resources in the healthcare sector, while leading to the interruption of the typical face-to-face contact between women and healthcare workers, has led to the identification of innovative methods for telephone or online support.

In October 2020 a second survey by UNICEF-UK through the NIFN on 135 healthcare workers highlighted how breastfeeding had a better outcome and greater user satisfaction in the Obstetrics departments, where in the meantime new methods of tele-support had been introduced (by telephone or video or in groups). Interviewees highlighted how some innovative services introduced during the pandemic period, including the TSB, can be maintained even after the pandemic, although virtual support should accompany – not replace – in-person support.

In general, published studies on TSB are essentially represented by a series of heterogeneous remote interventions by target population (mainly in complex or disadvantageous situations), delivery method and intensity of remote contacts [15–17]. Despite the limitations of this heterogeneity, the effectiveness of TSB is confirmed by the systematic reviews of Hubschman-Shahar [18] and Gavine [19]. Particularly, Gavine shows that remote support increases exclusive breastfeeding rates 3 months after birth by 25% compared to standard assistance and can have a positive effect even up to 6 months.

Finally, we must recognize that TSB is highly appreciated by user satisfaction (parents) as well as professionals [20].

## Remote support to breastfeeding versus TSB

The remote support in breastfeeding uses different methods, although in most cases it involves a simple written transmission (via email or WhatsApp) of a response given to mother [21]. Such experiences, characterized by asynchronous consultancy, although relevant in terms of the amount of activity, effectiveness and satisfaction on the part of women, are not the subject of this document as they lack any visual contact between the health worker and the mother.

The Breastfeeding Section of the SIN conducted an online survey on TSB between October 30th and December 18th, 2023. This study explored the experience on TSB in Maternity Hospitals (MHs) with NICU. The results are based on responses from 50 out of 110 NICUs, which accounted for 45.4% of the total. Interestingly enough, MHs provided no synchronous TSB and, unexpectedly, only 15 out of 50 MHs (30%) provided some support, that simply consisted of a telephone call (Table 1). Actually, in the context of the present paper, we do not consider the support provided via telephone by the above mentioned 15 NICUs as a type of TSB, not being paired with a video-visit.

**Table 1:** Remote support in breastfeeding in 50 Italian MHs with NICU

	MH with NICU (N)	Percentage (%)
• Yes, with video contact	0	0
• Yes, just a telephone call without video contact	15	30
• No, but TSB was active in the past	0	0
• No, but we plan to activate TSB	7	14
• No, but it does exist in our community	4	8
• No	24	48
Total	50	100

## Italian experiences on the TSB

We report a selection of recent experiences on the TSB made in Italy, according to the definition given by the SIN.

### Health centers

During the pandemic, between year 2019 and 2021, the Health Centers of the Baby Friendly Community ASUGI in Trieste succeeded to give online assistance to a stable percentage (around 60%) of pregnant women, who would then give birth at the Maternal-infant Institute IRCCS Burlo Garofolo, the only Maternity Hospital of the Province of Trieste. In these settings, the TSB provided by midwives has proven effective to keep at around 51% the rate of exclusive breastfeeding at 4–5 months of life [22].

### A qualitative study in the NICU

The SIN in collaboration with the Italian Society of Neonatal Nursing (SIN-INF) conducted a qualitative study with the methodology of Focus Group (FG) on the TSB.

FG implies a group discussion on a specific topic, allowing members to verbalize unaware, latent or otherwise difficult to emerge elements.

The study aimed to explore the TSB before, during and after the pandemic. It sought to identify strengths and weaknesses, strategies implemented, experiences of staff and families and suggestions for future improvements provided by the participants.

Two 90 min FGs, with 11 NICU nurses and 10 neonatologists respectively, were conducted by two facilitators in May 2022 (Table 2) [23]. A convenient sample of Italian NICUs was selected. Participants were individually recruited, homogeneous by profession, with varying levels of commitment to breastfeeding. To activate the discussion in the FGs, the facilitators proposed a semi-structured questionnaire to the participants (Table 3).

**Table 2:** Characteristics of nurses and neonatologists, who participated in the FGs

Characteristics of participants to FGs	Nurses (N: 11)	Neonatologists (N:10)	Total (N: 21)
Age (years): mean (range)	44 (31–59)	46 (35–61)	45 (31–61)
Sex			
• Male n (%)	3 (27)	-	3 (14)
• Female n (%)	8 (72)	10 (100)	18 (86)
Education			
• Three-year degree n (%)	7 (64)	-	7 (33)
• Five-years degree n (%)	4 (36)	-	4 (19)
• Medicine degree n (%)	-	10 (100)	10 (48)
Profession			
• Nurse n (%)	7 (64)	-	7 (33)
• Pediatric Nurse n (%)	3 (27)	-	3 (14)
• Nurse Coordinator n (%)	1 (9)	-	1 (5)
• Physician n (%)	-	10 (100)	10 (48)
Postgraduate degree			
• Specialization n (%)	-	10 (100)	10 (48)
• Master n (%)	4 (57)	3 (30)	7 (33)
• PhD n (%)	-	2 (20)	2 (9)
• Other degree n (%)	3 (43)		3 (14)
Level of Hospital Care			
• Hub n (%)	8 (73)	7 (70)	15 (71)
• Spoke n (%)	-	2 (20)	2 (9)
• Others n (%)	3 (27)	1 (10)	4 (19)
Type of hospital ward			
• NICU	9 (82)	6 (60)	15 (71)
• Sub Intensive Neonatal Unit	1 (9)	-	1 (5)
• Special Care Nursery	-	3 (30)	3 (14)
• Other	1 (9)	1 (10)	2 (9)
Lenght of service			
• Years: mean (range)	20 (10–39)	17 (5–28)	19 (5–39)
Lenght of service in Neonatology			
• Years: mean (range)	17 (6–39)	14 (4–28)	16 (4–39)

**Table 3:** Questions asked to participants in the 2 FGs on TSB

a) Do you think that support for breastfeeding still requires at least some in-person contact? or do you think that TSB can effectively complement in person assistance?
b) Do you believe that TSB can be effective?
c) Do you think it is possible to implement TSB in your facility?

The story telling of the 21 participants in the 2 FGs was audio-recorded, transcribed, anonymized and analyzed for predefined categories as well as for emerging categories. Participants described the different types of support applied in their work environments and with which they had experience/knowledge. The comments listed below emerged in one or both of the two FGs.

#### Main comments from both the nurses and the doctors’ FGs

- A lack of sufficient breastfeeding support in some MHs was already reported before the COVID-19 pandemic. Consequently, providing TSB during an emergency situation with preexisting sub-optimal support was particularly challenging.
- The implementation of TSB had to deal with the chronic and economic shortage of human and material resources. This explains why during the initial phases of the pandemic, given the limitations of access to the hospital, in some cases the TSB was rather provided by local services or by peer volunteers.
- Understandably, it is more difficult to communicate, be empathetic, and provide relevant help to mothers during a remote contact, instead of an in-person visit.

#### Main comments from the nurses’ FG

- The TSB has been usually included in the routine general care.
- The NICU nurses emphasized that even before the pandemic, the hospital used to care for newborns who had been discharged from the NICU and their mothers, at least through telephone consultations. A strong and continuous relationship is usually established between the hospital and the families as part of the follow-up service.
- For successful breastfeeding support, health staff must have a positive attitude towards the presence of parents in Postnatal Wards and NICUs. In fact, in settings where this is not the case, the TSB would paradoxically become a tool to perpetuate the separation between parents and their newborns. In many MHs, fathers were excluded from accessing rooming-in areas and NICUs for too long after the end of the pandemic. In some NICUs, the availability of video cameras in the unit, which allow parents to see their babies from a distance, appeared to influence the delay in parents being readmitted to the NICU.
- Midwives mostly dealt with the TSB, less often NICU nurses, and rarely doctors. Basically, the TSB was on a voluntary basis as institutional intervention was often lacking. Moreover, instead of receiving smartphones and/or tablets from the administration, most often the devices were personal or purchased from parents’ associations connected to the NICU (Table 4).

**Table 4:** Smartphones for the TSB

*«…We were available on call in the first few days … but we did not establish a real service for tele-support…»;*
*«…a smartphone was made available by parents' associations, who paid the contract with the phone operator…»;*
*«…the smartphone belonged to a doctor who gave it to us and we use it in a dedicated way»;*
*«… we have the Foundation's Wi-Fi and smartphone for the video calls”.”*

e. The staff in charge at the TSB did not receive appropriate training in telemedicine. Their selection was simply based on having attended a WHO Counseling Course on Breastfeeding. The need for health workers to acquire new skills to provide adequate online support through various and sometimes novel devices was not taken into account. Consequently, when the TSB was implemented, the staff had to familiarize themselves with the devices mostly independently. However, this was not considered entirely negative, as competence is an asset that will endure.
f. At least for physiological newborns, the TSB should be organized by the community services, in the context of effective integration with the MH.
g. Although healthcare personnel were sometimes worried by the new experience, the TSB as a whole was considered positively both for the novel mode of supporting mothers and for the rewarding prompt and generous response of the staff in organizing the service (Table 5).

**Table 5:** The individual initiative of health workers for the TSB

*«We learned to use the tablet to make video calls with parents who couldn't come to hospital. And this has remained in certain way even now…";* *“…a great will and great individual initiative in supporting mothers"*

#### Main comments from the doctors’ FG

- Even in cases where the pre-pandemic breastfeeding support was inadequate, we must acknowledge that some important steps have been taken, particularly by dedicated midwives focused on postpartum care and breastfeeding.
- Given staff shortage, health workers engaged with the TSB did so adding this to other routine activities.

Summarizing, the FGs witnessed the great variability of the TSB in Italian MHs with regards to type of device (smartphone, tablet computer), owner of the device (property of the department/foundation rather than health professionals) and mode of access (e.g.: free access during a dedicated time window or following the request of the mother or as part of the NICU follow-up).

The experience with the TSB was challenging and, although it caused some confusion and discomfort among the staff, overall, it was satisfactory for both health workers and families (see Table 6). In particular, the nearly constant presence of fathers at the TSB activities during the lockdown was evaluated as positive by interviewees. On the contrary, the lack of a timely availability of cultural mediators represented an obstacle for the TSB, when directed to foreign people.

**Table 6:** Families and the TSB

*The TSB was «Much appreciated by mothers, especially in the first part of the lockdown, when they were truly alone»…*	

Finally, the FG participants suggested that the TBS should be complementary to the in-person support, that still remains indispensable (Table 7), and that it should be organized as an integrated service between the community health services, voluntary associations and the MH. This is particularly needed when considering the current staff shortage.

**Table 7:** An obvious limitation of the TSB: lack of in presence contact

*«Effective support for breastfeeding also requires presence when we are unwell, we also need a shoulder to lean on from our loved ones…so the use of telemedicine scares me...»*	

### The Milan COD20 IT platform

The TSB was promoted during the COVID-19 pandemic by the corporate ASST Fatebenefratelli-Sacco in Milan, Lombardy. The COD20 IT platform (acronym for Hospital Care at Home; https://www.cod20.it/), originally dedicated to hospital care during the COVID-19 pandemic [24], has been adapted for 7 Health Centers that were enabled to provide support for the management of breastfeeding (e.g.: observation and assessment of latch and of breast diseases during lactation) as well as to inform groups of women during pregnancy and postpartum. Breastfeeding women with severe issues (e.g.: mammary abscess) were addressed to the hospital and on the contrary, women who were identified during the hospital stay to suffer from mental disease (such as postpartum depression) were referred for treatment to the community mental health services.

During the pilot phase the support of a project manager was crucial, helping the operators understand the system’s potential and address critical issues that arose from its use.

Finally, the platform was fully functional in February 2021. No Apps are used, nor registration on the portal is required: the system is accessible via browser from any device. Remote visits, consultations, clinical reports and medical prescriptions can be consulted in the medical dossier created by the patient or in the electronic health record. Following a remote assessment, the specialist can decide for an in person visit.

The TSB project via COD20, called Telelactation©, has achieved considerable appreciation both from mothers, particularly those who live far from services, and from operators, especially younger ones. In 2022, more than 500 mothers received remote support, in addition to over 1500 home visits.

## Strengths and limitations of the TSB

When planning for the implementation of the TSB, we should take into account feasibility, accessibility, functionality, effectiveness and economic-financial implications [25]. In other words, we should be aware of the advantages and the limitations of the TSB, consistently reported in the UNICEF-UK experience as well as in the above FG study reported for Italy by SIN and SIN-INF.

### Strengths

A first, immediately appreciated characteristic of the TSB is the overcoming of barriers relating to time and space with a prompter response in providing assistance. This greater accessibility of women may prove to be essential when in-person visit is not possible, particularly for some mothers, who do not feel ready to leave home or have no available transport [25]. Moreover, TSB may lead to economic savings due to reduction of direct and indirect costs, a lower environmental impact and possibly a greater user satisfaction.

Secondly, women can be observed in their environment (so that advice can be better focused on the family context) and/or possibly outside normal working hours with a higher chance for the partner to be present and involved.

Thirdly, the hands-off technique, implicit in video consultation, could increase the mother's confidence in her own ability to breastfeed.

Fourth, integrating the TSB among community health services, mental health services and MHs can improve not only the effectiveness and quality of care provided, but also the relationships between professionals in different sectors of the healthcare system.

Lastly, virtual support may reduce the time for home visiting staff, as no travel is required, thus allowing more women to be seen.

### Limitations

The TSB obviously implies an altered relationship between the health worker and the woman, that may question the feasibility of an empathic communication. Unluckily, training to manage the TSB is provided inconsistently and, consequently, a major barrier encountered in providing an adequate service may be staff incompetence and reluctance, that worsens any pre-existence staff shortage.

Other barriers to the TSB may be technology-related, such as the unavailability of updated specific operating systems inside health services, the impending interruption of the online connection for technical reasons and the digital divide between different users. The last is usually due to limited access to internet, unequal poor digital skills (for example between Baby Boomers and Millennials) [27] and non-homogeneous geographical availability of broadband [28].

Finally, for their part, women may just not have been aware of the availability of TSB or may believe that the remote support method do not adequately meet their needs.

## Regulations and financing of the TSB in Italy

In order to avoid a legislative and regulatory backwardness in the face of a rapid technological innovation, in 2020 the Italian National Institute of Health issued the operative indications on Telemedicine [29, 30], followed in 2022 by two ad hoc decrees of the Minister of Health and the Minister of Technological Innovation and the Digital Transition which issued the Guidelines for telemedicine services [31, 32].

As part of Mission 6-Health, in the National Recovery and Resilience Plan [33], telemedicine is now recognized as playing a central role in the reorganization of the community’s individualized care. Particularly, a national telemedicine platform has been planned, to be designed, implemented and managed by the National Agency for Regional Health Services (Agenas) [34].

Consequently, dedicated funds were allocated for the national telemedicine platform and for the Territorial Operations Centers (COT; one for each Health District) in order to overcome current technological non-homogeneity shown by the national mapping [35].

In other terms, according to the current legislation, telemedicine (and consequently the TSB) must use a platform approved by the health authority and which guarantees the privacy of the conversation and the identification of the service operators.

## Legal issues

According to Art 7, paragraph 2 of Law 24/217 (the so-called Gelli-Bianco Law) [36] the healthcare facility is also liable for «health services carried out… through telemedicine». Consequently, all legislative and ethical rules specific to healthcare professions apply also to telemedicine activities. Thus, telemedicine (and consequently also the TSB) activities imply the assumption of full professional responsibility (including data processing according to privacy) as for any other professional healthcare act, taking into account the limitations due to physical distance [29].

It should also be remarked that health insurance does not cover risks related to hardware/software defects and productions resulting from artificial intelligence (AI).

## The position statement of the SIN on the TSB

### Background

- The International health organizations (WHO, UNICEF) underline the importance of the support to breastfeeding, to be associated with its protection and promotion (https://www.who.int/westernpacific/activities/protecting-supporting-and-promoting-breastfeeding) and recognize the value of telemedicine [2, 26], including the TSB (https://www.unicef.org.uk/babyfriendly/guidance-documents/) [13].
- The TSB is a healthcare technology that has spread during the COVID-19 pandemic, but has showed to be effective and appreciated by users also outside emergency situations, with a good cost/benefit ratio.
- There is no precise and complete review on the TSB activities in Italy. However, there is a long-established remote support to breastfeeding provided by voluntary (LLLI) and professional (IBCLC) lactation consultants. Moreover, some hospital and community health services, particularly Baby Friendly Hospitals and Community (BFHI/BFCI) have been provided the TSB during the COVID-19 pandemic [22].
- Recent Italian Laws have defined the functional needs of telemedicine and the types of healthcare provision (in particular tele-visits and tele-consultations), but to date the expected regional organization and national or regional platforms dedicated to telemedicine are not yet available.
- In principle, community health services have the institutional mandate to support breastfeeding, even remotely, giving continuity of care along pregnancy, childbirth and postpartum. Nevertheless, the Obstetrics & Gynecology Department and the Neonatology/NICU, where deemed appropriate, might give their contribution to the organization and management of the TSB for the population of healthy term newborn, in an integrated manner with the community health services. Instead, the MHs with a NICU should provide the TSB for newborns with special needs as part of the existing follow-up activities.
- It seems appropriate, in case of possible implementation of the TSB activities by the Neonatology Wards and NICUs, to provide some brief indications, based both on the scientific evidence produced by the SIN (the 2022 Focus Groups with health professionals and the 2023 Survey in the NICUs), and on the experiences and surveys of UNICEF-UK.

### Indications from SIN on the TSB

**Fig. 1:**
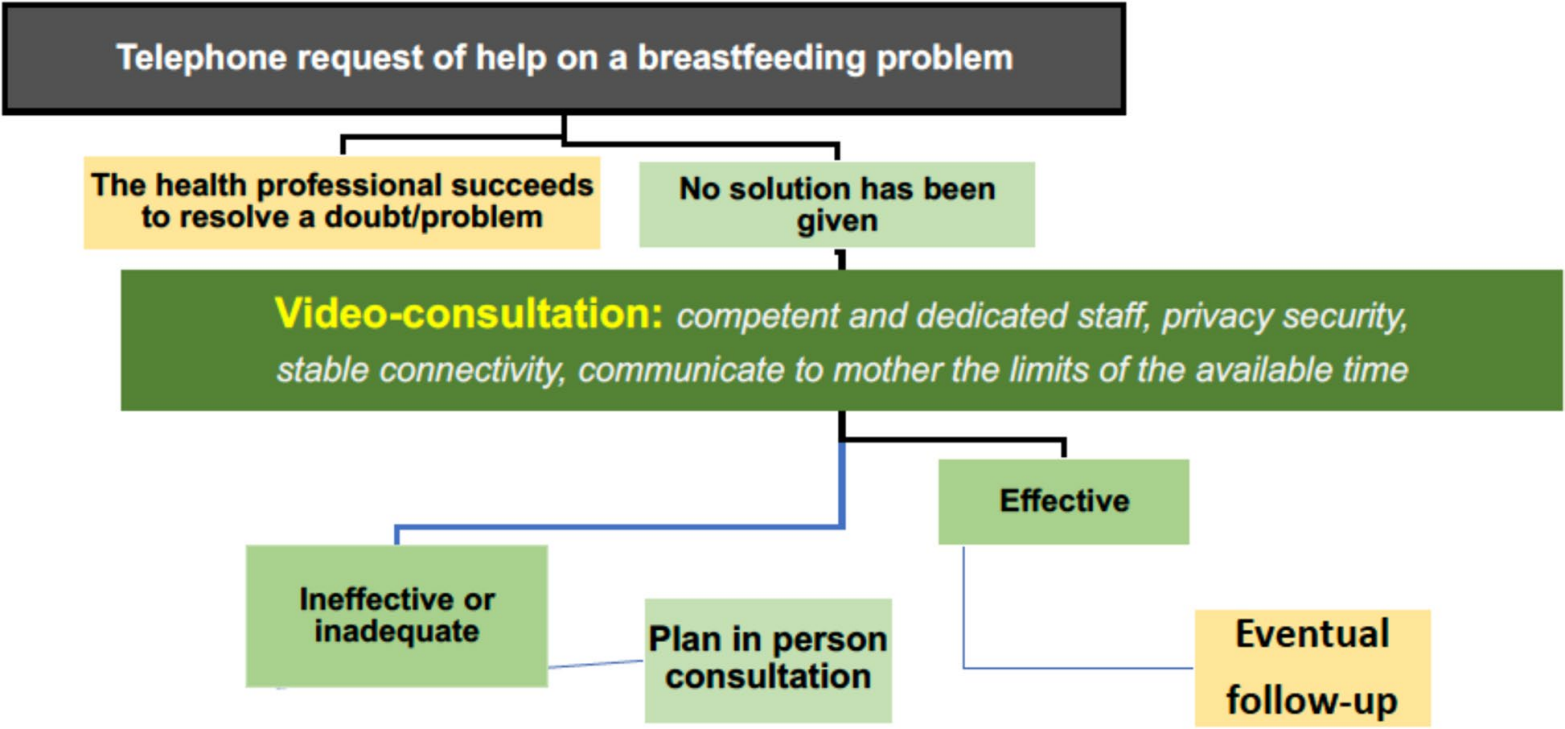
Operative flow chart proposed by the Italian Society of Neonatology for the tele-support in breastfeeding. Following a telephone request of help by the mother, a video-consultation may be provided. In person consultation might follow an ineffective or inadequate video-consultation

The SIN, limitedly to individual TSB, provides the following indications:

- Telephone contact with the user remains the first method of remote breastfeeding support and can allow triage for scheduling a video call, whenever possible even on the same day (Fig. 1).

- The TSB can be used when a consultation in person is not shortly available and may allow to select those situations that require an in-person visit (for example when the help, a woman needs, is complex).
- The TSB should be intended as a complementary method to the in-person assistance, that may increase the accessibility to health care.
- The TSB session requires a specific organization (https://www.unicef.org.uk/babyfriendly/guidance-documents/) which includes the use of competent healthcare staff (specifically trained and/or with adequate experience) and dedicated to the TSB, at least by time slot, and an appropriate methodology to prepare, run and conclude the support session (Table 8).
- Finally, according to current knowledge and policies, the TSB should be implemented and integrated in public health care services, while future research should more deeply explore its experience and impact in different settings.

**Table 8:** Practical hints for health workers (including neonatologists and nurses) about the individual TSB session, according to the indications from UNICEF-UK (https://www.unicef.org.uk/babyfriendly/guidance-documents/) modified by SIN

*1. Preparation of the TSB session*
a. assess the breastfeeding history and documentation before the video call;
b. plan each meeting, estimate the necessary time, agree with the mother the time limits of the TSB session;
c. make available the necessary assessment tools during the video call (e.g.: growth charts, breastfeeding assessment tool, protocols for the most common problems encountered during lactation);
d. choose an environment that guarantees the mother’s confidentiality;
e. respect the privacy requirements of the Italian Data Protection Authority (https://www.garanteprivacy.it/regolamentoue)^a^; send the mother the privacy form, agreed with the data protection officer (DPO);
f. check and ensure stable connectivity
*2. Running the TSB sessions *
a. introduce yourself, ask the mother to confirm the consent to carry out the video call and remind the mother of the maximum time available for the video call;
b. use an appropriate sequence of questions as a guide in counseling (e.g. number and duration of feedings, diuresis, pain during feeding, attitude of partners and family members towards breastfeeding);
c. support the mother by listening and asking for clarification. Explain what you have understood about the situation;
d. if unsure about a situation or unable to answer a question (e.g. use of a drug while breastfeeding), communicate this to the mother and then discuss it with an expert and call the mother back;
e. before ending the video call, ask the mother if she has any additional question;
f. plan eventual follow-up and/or referral (for example to a Breast Unit).
*3. Following the TSB session*
a. Record the relevant elements of the video consultation allowing identification of the person/patient and of the healthcare provider. Update any health card.
b. Organize mother referral to other professionals, if appropriate (e.g.: involvement of the Breast Unit in case of breast abscess).

^a^The GPDP is an independent administrative authority established by the so-called privacy law (Law No. 675 of 31 December 1996) and regulated subsequently by the Personal Data Protection Code (Legislative Decree No. 196 of 30 June 2003) as amended by Legislative Decree No. 101 of August 10, 2018

## Conclusion

According to the SIN, the TSB should be provided in an integrated manner by the community health services and the Maternity Hospital, being an effective intervention to promote breastfeeding as a complementary method to the in-person assistance.

## Data Availability

The datasets used during the current study are available from the corresponding author on reasonable request.

## Abbreviations

BFCI: Baby-Friendly Community Initiative
BFHI: Baby-Friendly Hospital Initiative
COVID-19: COronaVIrus Disease 19
MH: Maternity Hospital
NICU: Neonatal Intensive Care Unit
SIN: Italian Society of Neonatology
SIN-INF: Italian Society of Neonatal Nurses
TSB: Tele-support in Breastfeeding
UNICEF: United Nations International Children's Emergency Fund
WHO: World Health Organization
